# Structural Characterization of Chondroitin Sulfate from Hybrid Sturgeon (*Acipenser schrenckii* × *Huso dauricus*) Cartilage and Its Alleviating Effect on Osteoarthritis

**DOI:** 10.3390/nu18101494

**Published:** 2026-05-08

**Authors:** Shanshan Zhang, Yanyan Li, Mingxiao Yu, Xue Zhao, Zeyu Liu, Tingting Yang, Changwei Wang, Hu Hou

**Affiliations:** 1 State Key Laboratory of Marine Food Processing & Safety Control, College of Food Science and Engineering, Ocean University of China, Qingdao 266000, China; zss010315@163.com (S.Z.); liyanyan3700@163.com (Y.L.); yumingxiaoouc@163.com (M.Y.); zhaoxue@ouc.edu.cn (X.Z.); lzy41652026@163.com (Z.L.); yangtingting@ouc.edu.cn (T.Y.); 2 Laboratory for Marine Drugs and Bioproducts, Qingdao Marine Science and Technology Center, Qingdao 266237, China; 3 Marine Biomedical Research Institute of Qingdao, Qingdao 266701, China; 4 Sanya Oceanographic Institution, Ocean University of China, Sanya 572024, China

**Keywords:** chondroitin sulfate from sturgeon cartilage (S-CS), sulfation pattern, structure-activity relationship, 16S rRNA, NF-*κ*B pathway, p38 MAPK pathway

## Abstract

**Objectives**: Given that the structure-activity relationship between sturgeon chondroitin sulfate (S-CS) and the alleviation of osteoarthritis (OA) remains unclear, we characterized the structure of S-CS and explored the relationship between its structure and its effect in alleviating OA. **Methods**: Chondroitin sulfate was extracted from sturgeon cartilage by alcohol precipitation. Its structure was thoroughly characterized using infrared spectroscopy, pre-column derivatization, high-performance liquid chromatography with PMP (PMP-HPLC), nuclear magnetic resonance spectroscopy (NMR), and other techniques. A rat OA model was established to explore the mechanism underlying its alleviation of OA. In addition, 16S rRNA sequencing was performed to investigate the role of gut microbiota. **Results**: S-CS was identified as a sulfated polysaccharide with an average molecular weight of 68.81 kDa and a GlcUA-to-GalN molar ratio of approximately 1:1. NMR analysis confirmed its characteristic 6-/4-sulfation patterns. Oral administration of S-CS at 100 mg/kg/d significantly alleviated joint damage by inhibiting the NF-*κ*B and p38 MAPK signaling pathways. Specifically, S-CS decreased the levels of p65 and p38 by 18.94% and 52.40% (*p* < 0.05), respectively, and decreased TNF-*α* concentration. Moreover, 16S rRNA sequencing showed that S-CS enhanced the diversity and richness of gut microbiota and reconstructed the microbial community structure. **Conclusions**: S-CS may be an effective supplement for OA.

## 1. Introduction

Chondroitin sulfate (CS), a member of the glycosaminoglycan (GAG) family, is an anionic polysaccharide with a linear conformation and a sulfated functional group [1,2]. The compound consists of repeating disaccharide units, which can be represented as follows: →4) *β*-D-GlcA (1→3) *β*-D-GalNAc [3]. CS can be classified into various types based on the location and degree of modification of the sulfate group, with types A (C4-sulfation) and C (C6-sulfation) [4,5,6]. 6-O-sulfate groups can enhance the inhibitory effect of CS on the NF-*κ*B signaling pathway, thereby downregulating the expression of inflammatory factors. In contrast, 4-O-sulfate groups promote chondrocyte proliferation, inhibit the expression of MMP-13 and ADAMTS4, and further protect the extracellular matrix (ECM). CS-A is predominantly found in mammalian cartilage, whereas CS-C is mainly distributed in marine species [7]. The benefits of CS have been demonstrated in anti-inflammatory [8], antioxidant [9], antitumor [10], and neuroprotective [11].

Commercially, CS is mainly derived from by-products from the processing of chickens, porcine, and bovine, with cartilage serving as the principal raw material [12,13]. Given the safety and religious concerns associated with these animal sources, CS derived from chicken and bovine cartilage may pose a cross-infection risk, such as bovine spongiform encephalopathy (BSE), H7N9 avian influenza, and other food chain crises [14]. In addition, Islam prohibits the consumption of porcine and porcine-derived products. Consequently, marine organisms, particularly fish, have gained attention as sustainable and alternative biological sources for CS production. Sturgeon, one of the most ancient extant bony fish families, contains approximately 10% cartilage relative to its body weight [12,15], and can be used to extract CS. Meanwhile, compared with terrestrial animals, the CS derived from sturgeon cartilage uses processing by-products as raw materials, which belongs to resource reuse. Moreover, sturgeon is a cultured species, involving no protection of endangered species and lower ethical controversy. CS extracted from sturgeon has been confirmed to have multiple activities. CS derived from hybrid sturgeon cartilage exhibits a range of biological activities [16]. CS purified from *Acipenser schrenckii* cartilage demonstrated significant cell proliferation-promoting activity [17]. CS extracted from hybrid sturgeon demonstrated the ability to restore gut microbiota homeostasis in mice with colorectal cancer [18], but few studies have investigated the therapeutic potential of sturgeon-derived CS in alleviating OA.

OA is a common chronic degenerative joint disease characterized by subchondral bone remodeling, synovial inflammation, and progressive articular cartilage degradation [19,20]. OA has a high global prevalence, with projections estimating nearly 1 billion cases by 2050 [21]. The progressive destruction of cartilage ECM represents a central pathological feature in OA, mediated by pro-inflammatory cytokines, NO, and aberrant activation of PI3K/AKT, NF-*κ*B, and MAPK signaling networks [22,23]. Consequently, this results in chronic pain, joint dysfunction, and reduced quality of life. At present, non-steroidal anti-inflammatory drugs (NSAIDs) are the primary pharmacologic agents for OA [24]. However, prolonged NSAID administration has side effects, such as gastrointestinal, cardiovascular, and renal complications [25]. Therefore, identifying pharmacological interventions that can decelerate OA pathogenesis remains an urgent research priority [26]. The incidence of OA is significantly higher in females than in males, especially in postmenopausal females, where the decline in estrogen levels accelerates articular cartilage degeneration, leading to a marked increase in the risk of OA [27]. Currently, most studies use male rats, and there are relatively few analyses on drug efficacy in females. Therefore, this study used female SD rats to establish an OA model, which is more consistent with the pathological state of clinical female OA patients and provides more targeted experimental evidence for the drug treatment of clinical female OA.

Therefore, S-CS, which was isolated from the cartilages of sturgeon, was systematically examined through physicochemical characterization and structural elucidation. The sulfation pattern was analyzed by Fourier transform infrared spectroscopy (FTIR) and NMR. The anti-OA activity in vivo of S-CS was evaluated using a rat OA model. The underlying structure-activity relationship mechanisms were further investigated by qRT-PCR and ELISA to assess the expression of related signaling pathways and key targets. S-CS may be an effective supplement for OA.

## 2. Materials and Methods

### 2.1. Materials and Reagents

Sturgeon (*Acipenser schrenckii* × *Huso dauricus*) cartilage was purchased from Sturgeon Dragon Technology Co., Ltd. (Quzhou, China) Sprague-Dawley (SD) rats (female, body weight 200 ± 20 g, 8 weeks old, SCXK2022-0006) were provided by Pengyue Experimental Animal Breeding, Co., Ltd. (Jinan, China). Alkaline protease was supplied by Pangbo Biological Engineering Co., Ltd. (Nanning, China). ELISA kits were purchased from Kexing Commerce & Trade Co., Ltd. (Shanghai, China) and Calvin Biotechnology Co., Ltd. (Suzhou, China). All primers were synthesized by Sangon Biotech Co., Ltd. (Shanghai, China). HPLC-grade methanol was obtained from Thermo Fisher Scientific Inc. (Waltham, MA, USA), and HPLC-grade acetonitrile was purchased from Merck KGaA (Darmstadt, Germany). Sodium hydroxide, glucose, phenol, and anhydrous ethanol were purchased from Sinopharm Chemical Reagent Co., Ltd. (Shanghai, China). All others were of analytical grade.

### 2.2. Preparation of Sturgeon Cartilage Chondroitin Sulfate (S-CS)

S-CS was prepared using the ethanol precipitation method [28]. Sturgeon cartilage was first crushed into about 8 mm particles and washed by soaking in dilute saline, and then crushed to a particle size of 20 mesh using a multifunctional crusher (Chendao Machinery Equipment Co., Ltd., Guangzhou, China). Following immersion in a 0.2 mol/L NaOH solution, it was subjected to centrifugation at 12 h intervals to collect the supernatant. Subsequently, the supernatant was supplemented with 0.2% alkaline protease and hydrolyzed at pH 9.0 and a temperature of 55 °C. After centrifugation at 4265× *g* for 15 min, the supernatant was mixed with 95% ethanol, and the precipitate was collected by filtration following 12 h of alcohol precipitation. After freeze-drying using a vacuum freeze dryer (Martin Christ Gefriertrocknungsanlagen GmbH, Osterode am Harz, Germany) at −60 °C under a vacuum of 0.05 Pa for 48 h, sturgeon cartilage chondroitin sulfate was obtained and designated as S-CS.

### 2.3. FTIR of S-CS

S-CS and KBr were thoroughly mixed, finely ground, and compressed into a transparent pellet. FTIR was carried out using the NICOLET iS10 Fourier Transform Infrared Spectrometer (Thermo Fisher Scientific Inc., Waltham, MA, USA) with 64 scans over the wavenumber range of 4000–400 cm^−1^ [28].

### 2.4. Molecular Weight Distribution Analysis of S-CS

Sodium dodecyl sulfate polyacrylamide gel electrophoresis (SDS-PAGE) was used to determine the molecular weight distribution of S-CS [29]. A 5% stacking gel and 15% separating gel were prepared, with an electrophoresis voltage of 200 V applied for 27 min. Following electrophoresis, the gel was stained with a staining solution for 1 h and subsequently destained with a 2% (*w*/*v*) acetic acid solution until the gel background became transparent. The resulting electrophoretic gel was scanned using an EPSON scanner.

### 2.5. Analysis of the Molecular Weight of S-CS

High-performance size-exclusion chromatography (HPSEC) (Agilent Technologies Inc., Santa Clara, CA, USA) was employed to determine the molecular weight of S-CS [30]. The chromatographic column was OhPak SB-804HQ (8.0 mm × 300 mm, 10 μm). A mobile phase of 0.1 mol/L Na_2_SO_4_ was delivered at a constant flow rate of 0.4 mL/min. The oscillometric detector was selected.

### 2.6. Determination of Molecular Weight Distribution Changes in S-CS During In Vitro Simulated Digestion

The static simulated digestion in vitro was performed according to a previously reported method with minor modifications [31], including oral (O), gastric (G), and intestinal (I) stages. Oral digestion was done by adding 5 mL S-CS sample to 3.5 mL of simulated saliva (SSF), 0.5 mL of salivary amylase solution (1500 U/mL), and then CaCl_2_ solution (0.3 mol/mL) was added, and the pH was adjusted to 7, and then digested at 37 °C for 30 min. The gastric phase was initiated by adding 8 mL of the oral digest to 6 mL of simulated gastric fluid (SGF), 1.28 mL of pepsin solution (25,000 U/mL), and 4 μL of CaCl_2_ solution (0.3 mol/mL). After pH adjustment to 3.0, digestion proceeded at 37 °C for 6 h. Then in the intestinal phase, 6 mL of the gastric digest was mixed with 3.3 mL of simulated intestinal fluid (SIF), 1.5 mL of pancreatin solution (800 U/mL), 0.75 mL of bile salt solution (160 mmol/mL), and 12 μL of CaCl_2_ solution (0.3 mol/mL), followed by 6 h of digestion at 37 °C. During the gastric and intestinal digestion phases, samples were collected at 0, 0.5, 1, 2, 4, and 6 h, followed by enzyme inactivation for subsequent determinations.

### 2.7. Determination of the Monosaccharide Composition of S-CS

The monosaccharide profile of S-CS was determined using pre-column PMP derivatization coupled with high-performance liquid chromatography (PMP-HPLC) employing an Agilent chromatographic system (Agilent Technologies, Inc., Santa Clara, CA, USA). The chromatographic column was a Poroshell EC-C_18_ separation column (4.6 × 150 mm, 2.7 μm), and an ultraviolet detector was used for detection. The maximum column pressure was 200 bar, and the column temperature was 30 °C [32]. Nine monosaccharide standards, including Man, GlcN, GalN, GlcUA, Rha, Xyl, Glc, Gal, and Fuc, were prepared into 20 mmol/L standard solutions. A 10 μL aliquot of each solution was mixed with the internal standard Lac to a total volume of 100 μL, followed by the addition of NaOH and PMP derivatization reagent. The derivatization was conducted at 70 °C in the dark for 30 min. After adding chloroform, the mixture was centrifuged at 4000× *g* for 5 min, and 10 μL of the supernatant was filtered through an ultrafiltration membrane before injection. S-CS was prepared into a 10 mg/mL solution, and 200 μL of the solution was mixed with 1 mL of 2 mol/L trifluoroacetic acid in an ampoule. After flushing with nitrogen and sealing, hydrolysis was performed in an oven at 110 °C for 4 h. The residue was re-dissolved in ultrapure water and evaporated to dryness to remove trifluoroacetic acid, then neutralized with 0.3 mol/L NaOH and diluted to 200 μL. A 90 μL aliquot was mixed with 10 μL of Lac internal standard, and the subsequent procedures were identical to those used for the standard solutions.

### 2.8. Nuclear Magnetic Resonance Spectroscopy of S-CS

A 40 mg sample of S-CS was precisely weighed and lyophilized by dissolving it with 550 μL of D_2_O and exchanging it with D_2_O three times [33]. The lyophilized sample was dissolved with 550 μL of D_2_O, mixed, and centrifuged (4265× *g*, 15 min), and detected using a JNM-ECP 600M (JEOL Ltd., Tokyo, Japan) superconducting NMR spectrometer. 1D NMR spectra (^1^H and ^13^C) and 2D NMR spectra (^1^H-^1^H COSY and ^1^H-^13^C HSQC) were acquired. Data processing was performed using MestReNova 9.0 software.

### 2.9. Animals and OA Model

The rat experiments were approved by the Animal Experimentation Ethics Committee of the Ocean University of China (No. SPXY2023061230) on 12 June 2023. Sprague-Dawley (SD) rats (female, body weight 200 ± 20 g, 8 weeks old, SCXK2022-0006) were provided by Pengyue Experimental Animal Breeding. SD rat is a well-established and widely used model in OA research due to its stable genetic background, similar joint anatomy to humans, and consistent pathological manifestations that closely mimic human OA progression [34]. SD rats were housed for 11 weeks at a temperature of 24 ± 1 °C, receiving sufficient feed and water every day. After one week of acclimatization, according to the preliminary experiments, 64 rats were randomly divided into 8 groups, with 8 rats in each group. Except for the NC (normal control) and Sham (sham operation) groups, OA modeling was performed in all other groups and was surgically induced in the left knee joint through medial meniscectomy [35]. Specifically, the rats were anesthetized via intraperitoneal injection, an incision was made on the medial side of the left knee joint, the tissues were separated layer by layer, the anterior cruciate ligament was transected, and the medial meniscus was excised. After the operation, the joint cavity was rinsed with normal saline and sutured layer by layer. After the OA model was established, the low-dose group (SL group) was administered 20 mg/kg/d through oral gavage, and the high-dose group (SH group) was administered 100 mg/kg/d. The NC, Sham, and MC (model control) groups received normal saline via gastric gavage, while the PC (positive control) group was treated intragastrically with meloxicam (0.675 mg/kg/d), which is the most popular option. Serum and knee tissue samples were collected after surgery for 8 weeks.

### 2.10. Micro-CT of Rat Knee Joints

Whole-scan imaging was performed using a BRUKER Micro-CT SkyScan 1176 3D microtomography system (Shanghai Yinci Biotechnology Co., Ltd., Shanghai, China) to evaluate osteophyte formation and subchondral bone morphology, with the voltage of 65 kV and the current of 385 μA. Three-dimensional (3D) reconstructed images were acquired using a Micro-CT system. Meanwhile, the following parameters were calculated: bone surface area to bone volume ratio (BS/BV), bone surface area to tissue volume ratio (BS/TV), trabecular thickness (Tb.Th; mm), trabecular number (Tb.N; 1/mm), trabecular separation (Tb.Sp; mm), and bone mineral density (BMD) [36]. Among these indices, BS/BV and BS/TV were calculated following image segmentation; Tb.Th, Tb.N and Tb.Sp were determined using the bone morphometry module built into the software, and BMD was calculated via calibration against a standard bone mineral density phantom.

### 2.11. Histological Staining Analysis of the Rat Knee Joint

The rat knee joints preserved in 4% paraformaldehyde solution were made into paraffin sections [37], subjected to hematoxylin-eosin (HE), Van Gieson (VG) staining, and Safranin-O/Fast Green (S-O/FG) staining, and subsequently observed with light microscopy (Nikon Co., Ltd., Tokyo, Japan).

### 2.12. Immunofluorescence Staining of the Rat Knee Joint

The knee joint tissue was embedded in paraffin and made into 5 μm-thick sections. The sections were baked at 55 °C for 30 min, followed by dewaxing and antigen repair. Then, they were covered with 5% blank goat serum and sealed at 37 °C for 30 min [38]. After adding the primary antibody (BSA diluted 1:200), incubated overnight at 4 °C. After washing with PBS, added the secondary antibody (BSA diluted 1:400) and incubated for 1 h. After washing with PBS, the DAPI working solution was added and incubated for 10 min for nuclear staining. Finally, washed with PBS, and then added anti-fluorescence quenching mounting agent for mounting. A fluorescence microscope was used to capture the images. Semi-quantitative analysis of Mean fluorescence intensity (Mean) was performed using ImageJ 1.54f (National Institutes of Health, Bethesda, MD, USA).

### 2.13. Determination of Quantitative Real-Time Polymerase Chain Reaction (qRT-PCR)

Trizol reagent was used to extract total RNA from articular cartilage, followed by reverse transcription into cDNA. qRT-PCR was performed using a Line Gene 9600 Plus Fluorescence PCR Detection System (Bioer Technology Co., Ltd., Hangzhou, China). For qRT-PCR analysis, mRNA expression levels were determined using the 2^−ΔΔCt^ method [39] using specific primer sequences (Table S1).

### 2.14. Analysis of Cytokine Concentrations in Serum

Blood samples were collected and centrifuged at 8000× *g* for serum isolation. Serum factor concentrations were quantified using commercial ELISA kits (Shanghai Enzyme-linked biotechnology Co., Ltd., Shanghai, China).

### 2.15. Analysis of Intestinal Microbial Diversity

Gut microbiota diversity analysis was adapted from previously established methods [40]. The total DNA of microbial communities was isolated using a DNA extraction kit. To amplify the V3-V4 region of the 16S rRNA gene, a PCR assay was conducted with the forward primer 338F (5′-ACTCCTACGGGAGGCAGCAG-3′) and the reverse primer 806R (5′-GGACTACHVGGGTWTCTAAT-3′). PCR products were recovered, purified, and quantified using Qubit 4.0.

### 2.16. Statistical Analysis

Values are represented as means ± SD. Graphical and statistical analyses were performed using Origin Pro 2023 and IBM SPSS Statistics 26. Intergroup comparisons were assessed by one-way analysis of variance (ANOVA) and Duncan’s multiple range test, with statistical significance defined as *p* < 0.05.

## 3. Results and Discussion

### 3.1. Structural Characteristics Analysis of S-CS

**Figure 1:**
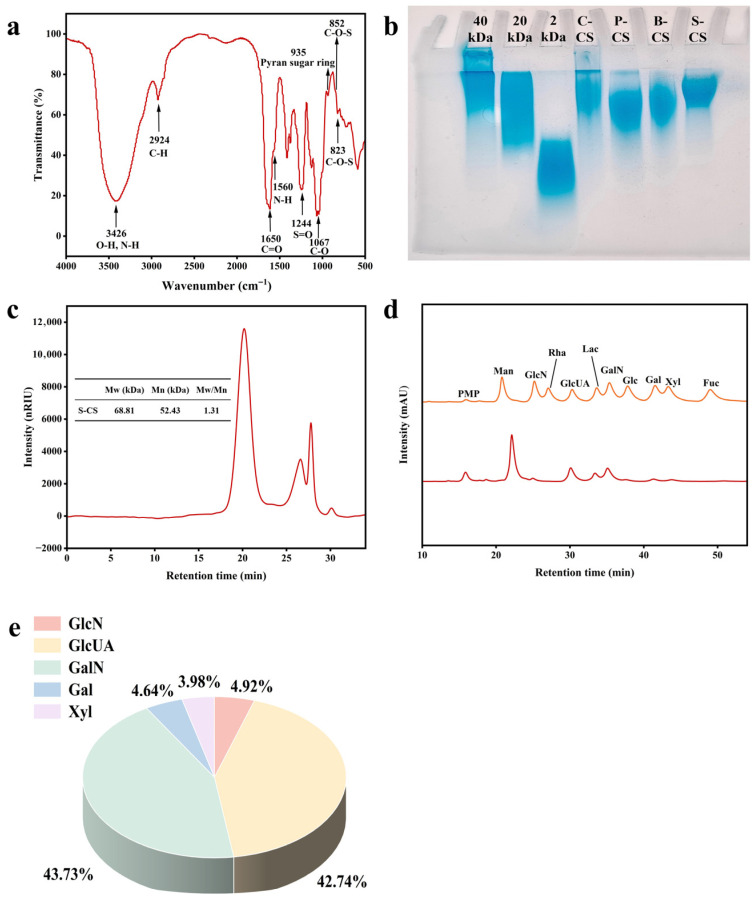
Structural Characterization and Physicochemical Properties of S-CS. (**a**) The FTIR analysis of S-CS, (**b**) The SDS-PAGE electrophoretic analysis of S-CS, C-CS, P-CS, and B-CS, (**c**) The HPSEC analysis of S-CS, (**d**) The PMP-HPLC analysis of S-CS, and (**e**) The Monosaccharide composition of S-CS (Orange line: standard monosaccharide, red line: S-CS sample).

The S-CS was prepared according to the procedure illustrated in the methods with a yield of approximately 16%. The structural features of S-CS were characterized. FTIR spectroscopy (Figure 1a) revealed a strong characteristic absorption band at approximately 3420 cm^−1^ in S-CS, corresponding to the stretching vibration of O-H, confirming the presence of hydroxyl groups typical of polysaccharide structures [41]. The characteristic absorption band observed around 2930 cm^−1^ correlates with the C-H stretching vibration. The peaks produced by the C=O stretching vibration and N-H variable-angle vibration are at 1650 and 1560 cm^−1^, indicating that there is an acetyl-amino structure in S-CS. The absorption peak at 1650 cm^−1^ corresponds to the amide I band of CS-C [40]. The peak produced by the S=O bond in the sulfate group is at 1240 cm^−1^, which conforms to the characteristic structure of CS. S-CS has absorption peaks at 852 and 853 cm^−1^, so it may contain A-type and C-type CS [13]. However, the resolution of infrared spectroscopy is low, and the accuracy is poor; it cannot accurately distinguish the substitution of sulfate groups, so it needs to further analyze the fine structure of chondroitin sulfate with the help of nuclear magnetic resonance spectroscopy.

The functional characteristics and biological efficacy of CS are significantly influenced by its molecular weight [42]. SDS-PAGE was used to estimate the molecular weight distribution of S-CS (Figure 1b). Dextran sulfate standards with average molecular weights of 40, 20, and 2 kDa, along with CS extracted from chicken cartilage, porcine cartilage, and bovine cartilage (designated as C-CS, P-CS, and B-CS, respectively), served as controls. S-CS was primarily distributed within the region of the stacking gel and exhibited a relatively concentrated band profile, with an estimated molecular weight range of 20–90 kDa. According to previous studies, CS isolated from terrestrial organisms typically exhibits lower molecular weights, within the range of 13 to 26 kDa, but CS derived from fish generally demonstrates a broader distribution [33]. S-CS with a molecular weight in the range of 20–90 kDa has better water solubility and bioavailability, is more stable in the intestinal tract, can be better absorbed into the blood, has stronger penetrability, can act on articular chondrocytes more efficiently, and inhibits inflammatory responses and ECM degradation [43].

As shown in Figure 1c, the molecular weight of S-CS is further characterized. Different sources and extraction methods affect the molecular weight. Wang et al. isolated CS with an average molecular weight (Mw) of 299 kDa from sturgeon cartilage [17,44]. However, the Mw value of CS isolated from the skull of a hybrid sturgeon is 38.5 kDa, and the bone-derived CS is 49.2 kDa [45]. The Mw values of S-CS were 68.81 kDa, and the dispersion coefficient was 1.31, indicating that the components were relatively uniform [46]. Generally, CS-0 units and CS-A units are present in higher abundance in CS polysaccharides from terrestrial animal tissues. Additionally, their Mw value is typically lower, ranging from 14.0 to 26.0 kDa, compared to marine-derived CS, which ranges between 30 and 70 kDa [47]. These findings indicate that CS molecular weight exhibits significant species-dependent variation.

The results of PMP-HPLC are shown in Figure 1d. The main composition of S-CS (Figure 1e) was glucuronic acid (GlcUA) and galactosamine (GalN), with a molar ratio of approximately 1:1, which is consistent with the typical monosaccharide composition of CS [30]. Studies have found that CS in the skulls of large hybrid sturgeon primarily consists of GlcN, GlcUA, GalN, and Gal, with a ratio of 0.7:4.9:4.8:1.0 [48]. In addition, S-CS contains small amounts of glucosamine (GlcN), galactose (Gal), and xylose (Xyl). Notably, the glucosamine content of S-CS reached 4.92%. The combined use of glucosamine and CS can alleviate rheumatoid arthritis in a rat model by modulating the intestinal flora [48].

### 3.2. Analysis of Simulated In Vitro and In Vivo Digestion Results of S-CS

**Figure 2:**
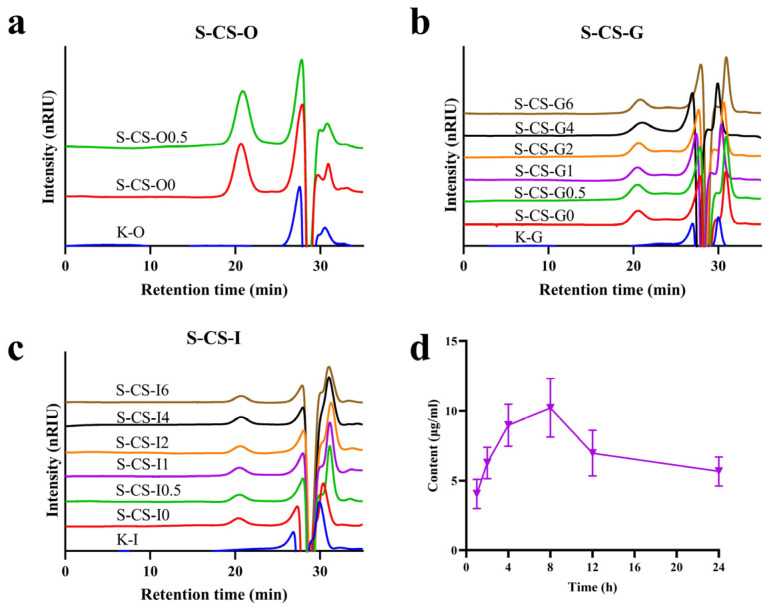
(**a**) The molecular weight changes in S-CS during the simulated oral digestion process (O represents the oral phase, K represents the blank digestive fluid, while 0 and 0.5 indicate different digestion times (h)), (**b**) Molecular weight distribution changes in S-CS during the gastric digestion phase (G represents the gastric phase, K represents the blank digestive fluid, and 0, 0.5, 1, 2, 4, and 6 denote different digestion times (h)), (**c**) Molecular weight distribution changes in S-CS during the intestinal digestion phase (I represents the intestinal phase, K represents the blank digestive fluid, and 0, 0.5, 1, 2, 4, and 6 denote different digestion times (h)), and (**d**) Serum S-CS concentration-time curve after CS oral administration in rats.

Further analysis was conducted on the molecular weight changes in S-CS after in vitro simulated digestion. The peak observed at 21.78–23.09 min was identified as a characteristic signal of CS. During the oral digestion phase, neither the response value nor the retention time of S-CS showed any significant changes (Figure 2a), indicating that its Mw remained unchanged. This suggests that *α*-amylase in the oral cavity had no degradative effect on S-CS. Studies have shown that certain polysaccharides containing *α*-(1→4) glycosidic bonds can be hydrolyzed by *α*-amylase in simulated saliva, while the polysaccharide chain of chondroitin sulfate is connected via *β*-(1→3) and *β*-(1→4) linkages, consistent with observations reported for oral digestion of fucosylated glycosaminoglycans from sea cucumber [49], rendering it resistant to oral digestion. Similarly, during the gastrointestinal digestion stage, no significant changes were observed at different time points (Figure 2b,c), further indicating that the Mw of S-CS did not decrease significantly and demonstrating that S-CS remains relatively stable throughout the digestive tract. These results suggest that CS is largely undecomposed when passing through the gastrointestinal system, which agrees with previous findings regarding the resistance of fucosylated CS from sea cucumber to degradation under salivary and gastrointestinal digestion [31]. The absorption efficiency of CS in rats was evaluated by monitoring the changes in concentration in serum (Figure 2d). The S-CS content increased continuously at 1 h after intragastric administration, and reached a peak value of 10.20 ± 2.09 μg/mL at 8 h, followed by a gradual decline back to the initial level. Massimiliano et al. [50] demonstrated via pharmacokinetic experiments on CS that CS can persist in joints for an extended period, thereby exerting long-term beneficial effects. These results indicate that CS within this molecular weight range can be absorbed into the rat body.

### 3.3. Nuclear Magnetic Resonance Spectroscopy

**Figure 3:**
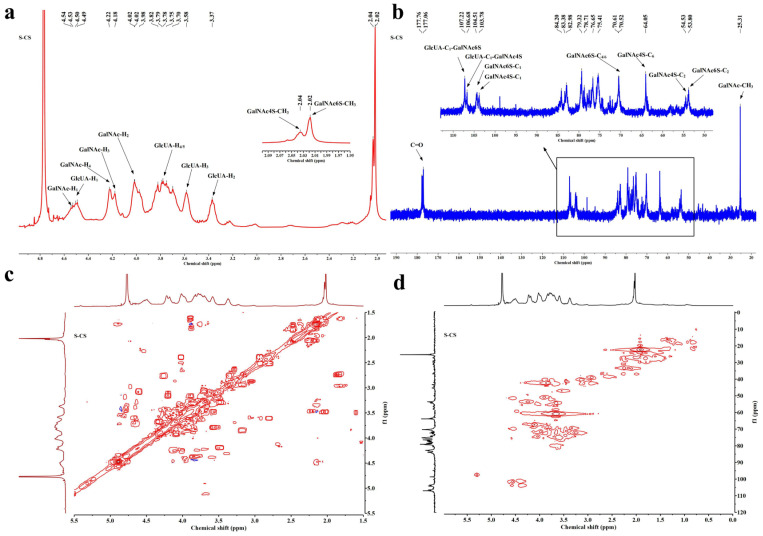
NMR spectroscopy and two-dimensional NMR hydrogen and carbon spectra analysis of S-CS. (**a**) The ^1^H NMR spectrum of S-CS, (**b**) The ^13^C NMR spectrum of S-CS, (**c**) The ^1^H-^1^H COSY of S-CS, and (**d**) The ^1^H-^13^C HSQC of S-CS.

The ^1^H NMR spectrum results are presented in Figure 3a. The signal attribution of S-CS is shown in Table 1. The ^1^H NMR spectrum of S-CS revealed that the signals at approximately 4.02 ppm and 4.54 ppm correspond to the H1 and H2 of GalNAc, respectively. The characteristic proton signals at 4.49 ppm (H1), 3.37 ppm (H2), and 3.58 ppm (H3) were clearly assigned to the glucuronic acid residue [51]. These chemical shift assignments are consistent with the monosaccharide composition analysis results. The results further demonstrate that the proton signals of S-CS all appear in two spectral regions of 2.00–2.10 and 3.00–5.00 ppm [52], which are the characteristic absorption regions of CS. S-CS showed peaks at 2.02 and 2.04 ppm, which are the signals generated by the methyl group on the CS acetyl group. The characteristic chemical shifts and signal patterns are indicative of both CS-C and CS-A repeating units in the sample [53]. This aligns with the results of FTIR spectrum analysis. S-CS displays strong signals at 4.18 (H6) and 4.22 (H4) ppm, corresponding to GalNAc6S. This observation aligns with the ^1^H NMR results of CS-4 and CS-6 in previous studies [52]. The characteristic peak generated by H1 of IdoA at 4.90 ppm did not appear in the atlas, indicating that there was no CS-B unit in S-CS. Previous studies suggested that the 6-O-sulfate groups can enhance the inhibitory effect of S-CS on the NF-*κ*B pathway, thereby reducing the expression of inflammatory factors [54]. In contrast, the 4-O-sulfate groups are capable of promoting chondrocyte proliferation, inhibiting the expression of MMP-13 and ADAMTS4, and further protecting the ECM [55].

The ^13^C NMR spectrum result of S-CS is shown in Figure 3b. The signal attribution of different sources of CS is shown in Table 2. In different ^13^C NMR spectra, apart from the signals of the *N*-acetyl methyl carbon (approximately 25.31 ppm) and the carbonyl group (around 177–178 ppm), the other signals of CS are primarily observed in the region of 50–110 ppm [12]. In the ^13^C NMR spectra of S-CS, the signals at 53.80, 70.52, and 104.51 ppm are related to C2, C4/6, and C1 of GalNAc6S, respectively, and the signals at 54.53, 64.05, and 103.78 ppm are related to C2, C6, and C1 of GalNAc4S, respectively. The signals at 106.68 (GlcA*β*1-3GalNAc6S) and 107.22 ppm (GlcA*β*1-3GalNac4S) are related to C1 [56]. These characteristic ^13^C NMR signals provide definitive evidence for the existence of both ΔDi-6S and ΔDi-4S disaccharide units within the S-CS sample. This structural assignment correlates with FTIR results.

The structure of S-CS was further validated through two-dimensional NMR analysis (Figure 3c,d). The two-dimensional nuclear magnetic resonance spectra confirm the presence of ΔDi-4S in S-CS, and the signal at *δ* H/C = 4.22/70.61 further validates the presence of ΔDi-6S in S-CS. In conclusion, S-CS possesses a unique 6-/4-sulfation pattern.

### 3.4. Effects of S-CS on Knee Joints in OA Rats

**Figure 4:**
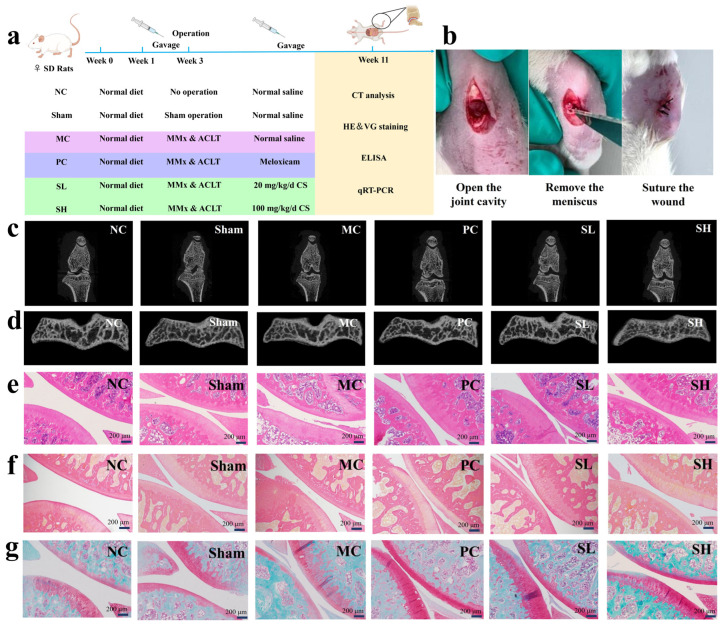
CT scan and histological staining of rat knee joints. (**a**) Animal experimental design, (**b**) Medial meniscus removal and ligament tear surgery, (**c**) Micro-CT scans of the rat knee joints, (**d**) Micro-CT scan of subchondral bone of rat knee joint, (**e**) HE staining (40×), (**f**) VG staining (40×), and (**g**) S-O/FG staining (40×).

The effects were evaluated using SD rats and the OA model while analyzing the differences between groups (Figure 4a). OA was successfully induced by medial meniscectomy and ligament tear in the left knee (Figure 4b) [35]. Micro-CT results showed significant differences in the knee joint structure among rats in different groups (Figure 4c,d). Compared with the MC group, the SL group still exhibited osteophytes (which remained relatively sharp) but no obvious cartilage defects; the SH group exhibited fewer osteophytes and no significant cartilage defects. Three-dimensional simulation reconstruction of the subchondral bone was performed, and the evaluation of bone morphological parameters is presented in Table 3. As shown in Table 3, compared with the NC group, the MC group exhibited decreased BS/BV, Tb.N, and BS/TV, as well as increased Tb.Sp, indicating destruction of subchondral bone in the MC group. In contrast, the SH group showed higher BS/BV, Tb.N, and BS/TV than the MC group, increased by 21.42%, 15.35%, and 15.45%, respectively, suggesting that the high-dose S-CS treatment can reduce bone loss and preserve the subchondral bone structure in OA rats. In summary, S-CS can reduce bone loss and protect the subchondral bone structure in OA rats.

The above results were further confirmed by histological staining. Through HE staining observation of knee joint tissues from each group (Figure 4e), it was found that the cartilage surfaces in the NC and Sham groups were smooth and intact, with chondrocytes arranged in a clear and organized manner. In contrast, the cartilage surface in the MC group appeared rough, and the chondrocyte number decreased. The cartilage surface in the PC group was smooth, with a clear structure and intact tide lines, but there were still local aggregations of chondrocytes. After S-CS intervention, the cartilage surface defects in each group showed improvement, with the high-dose group exhibiting more pronounced effects. Previous studies have demonstrated that CS-4 and CS-6 are considered to have chondroprotective effects, promoting the growth of bone and cartilage-related cells [47]. Therefore, S-CS could alleviate OA via a similar mechanism.

VG staining was employed to evaluate the organization and structural integrity of collagen fibers [53]. As shown in Figure 4f, the NC and Sham groups exhibited densely packed and well-organized collagen fibers on the cartilage surface, as well as a high collagen content. However, a significant reduction in collagen fiber content was observed in the MC group. The collagen fibers were neatly arranged, with a dense structure and a significantly wider distribution range in the PC group. Treatment with S-CS improved the disorganized arrangement of collagen fibers induced by OA, and the high-dose group demonstrated more pronounced improvements.

S-O/FG staining distinctly differentiated cartilage from bone tissues. As shown in Figure 4g, the NC and Sham groups showed smooth cartilage surfaces [56] and distinct cartilage-subchondral bone boundaries, with no differences observed between the groups. Relative to the NC group, the MC group had lighter, uneven Safranin staining and cartilage loss. Compared to the MC group, the low-dose S-CS group exhibited a significantly larger, uniformly stained safranin-positive area but narrowed cartilage spaces and persistent surface irregularity. The SH group displayed an expanded, deeper safranin-stained region with clear tidemarks, indicating a marked therapeutic effect.

### 3.5. S-CS Alleviates OA via Suppressing MMP-13/ADAMT4 and ECM Degradation

**Figure 5:**
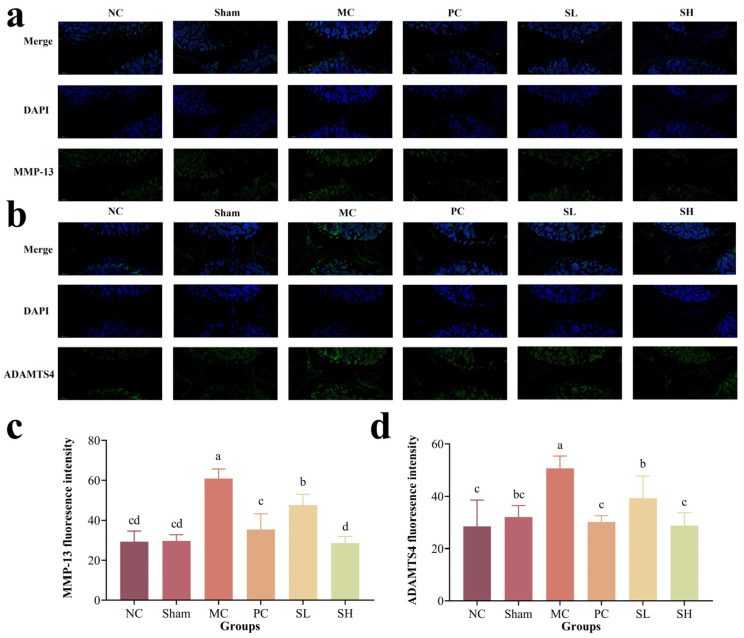
Immunofluorescence staining and semi-quantitative analysis. (**a**) Expression of MMP-13 in rat knee joint tissue, (**b**) Expression of ADAMTS4 in rat knee joint tissue, (**c**) Quantitative analysis of MMP-13 in rat knee tissue by immunofluorescence staining, (**d**) Quantitative analysis of ADAMTS4 in rat knee tissue by immunofluorescence staining (The presence of differing lowercase letters denotes significant intergroup differences, *p* < 0.05).

The extracellular matrix (ECM) is primarily regulated by the anabolic-catabolic balance in chondrocytes, and is critical for maintaining the normal physiological structure and biological function of articular cartilage. MMP-13 and ADAMTS4 are two important factors in the ECM. Studies have shown that MMP-13 impairs the synthesis of type II collagen and aggrecan [57,58], while ADAMTS4 also plays a key role in OA-associated cartilage degradation [59]. As shown in Figure 5a,c, Semi-quantitative analysis revealed a significant difference in MMP-13 fluorescence intensity between the MC and NC groups (*p* < 0.05); MMP-13 expression was markedly reduced in the PC group (*p* < 0.05). In the SL and SH groups, both the intensity and area of green fluorescence decreased to varying degrees, with semi-quantitative results confirming significantly lower MMP-13 expression (*p* < 0.05). Compared with the MC group, the fluorescence intensities of the SL and SH groups decreased by 21.69% and 53.04%, respectively. Consistent with the MMP-13 results, ADAMTS4 expression in the SL and SH groups was significantly decreased by 22.43% and 43.23% compared with the MC group (*p* < 0.05) (Figure 5b,d), supporting that S-CS alleviates OA via downregulating ADAMTS4. These results suggest that S-CS may exert its cartilage-protective effect by downregulating MMP-13 and ADAMTS4 expression. This aligns with Liu et al. [36], who found casticin reversed OA-induced MMP-13 upregulation. Therefore, it can be inferred that the alleviating effect of S-CS on OA is dose-dependent.

### 3.6. Role of S-CS in the Regulation of the NF-κB Pathway

**Figure 6:**
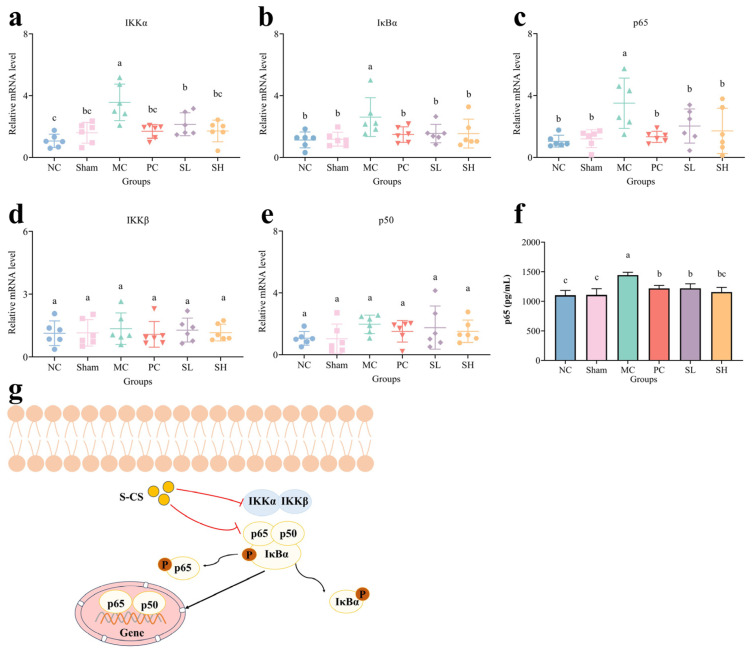
Analysis of relative mRNA levels and protein levels. (**a**) IKK*α*, (**b**) I*κ*B*α*, (**c**) p65, (**d**) IKK*β*, (**e**) p50, (**f**) The protein level of p65, and (**g**) NF-*κ*B signaling pathway diagram. (The presence of differing lowercase letters denotes significant intergroup differences, *p* < 0.05. The red lines in the mechanism diagram represent the conclusion obtained from experiments).

As shown in Figure 6a–c, qRT-PCR analysis revealed significantly elevated mRNA expression of IKK*α*, I*κ*B*α*, and p65 in the MC group (*p* < 0.05). IKK*α* contributes to the role of the IKK complex in regulating NF-*κ*B nuclear import [60]. qRT-PCR analysis revealed significant downregulation of IKK*α* mRNA expression in CS groups (*p* < 0.05). This suggests that S-CS may suppress the NF-*κ*B pathway and alleviate OA. mRNA levels of I*κ*B*α* and p65 were significantly down-regulated in the S-CS groups (*p* < 0.05) (Figure 6b,c). Studies have reported that OMT (Oxymatrine) exerts therapeutic effects on OA chondrocytes by downregulating NF-*κ*B activation through the reduction in p65 and I*κ*B*α* phosphorylation [61]. There are no significant differences in IKK*β* and p50 on the mRNA levels (*p* > 0.05) (Figure 6d,e). There was no significant difference in the expression level of p65 between the PC group and the MC group (*p* > 0.05). The SH and SL groups could downregulate the protein expression level of p65, with the average expressions decreasing by 286.78 and 222.11 pg/mL, respectively, relative to the MC group (Figure 6f). The decreased level of p65 may be associated with the inhibition of the NF-*κ*B signaling pathway. Guan et al. found that chondroitin sulfate (CS) can alleviate knee osteoarthritis by inhibiting the activation of the TLR4/NF-*κ*B pathway through analyzing the protein expression levels of p65 and p-p65 [56,62]. This result was consistent with the CT scan, where sustained activation of the NF-*κ*B pathway-mediated pro-inflammatory factors leads to osteophyte formation and articular cartilage surface roughness [63]. This suggests that S-CS may improve OA in rats by downregulating NF-*κ*B signal transduction. Previous literature has demonstrated that Δdi-4S and Δdi-6S disaccharides in CS can inhibit IL-1*β*-induced NF-*κ*B nuclear translocation in chondrocytes [64]. Given the presence of both Δdi-4S and Δdi-6S in S-CS, it is possible that S-CS functions via the NF-*κ*B signaling pathway. A definitive structure-activity relationship requires further validation using desulfated or fractionated derivatives. The possible signaling pathway is depicted in Figure 6g.

### 3.7. Role of S-CS in the Regulation of the p38 MAPK Pathway

**Figure 7:**
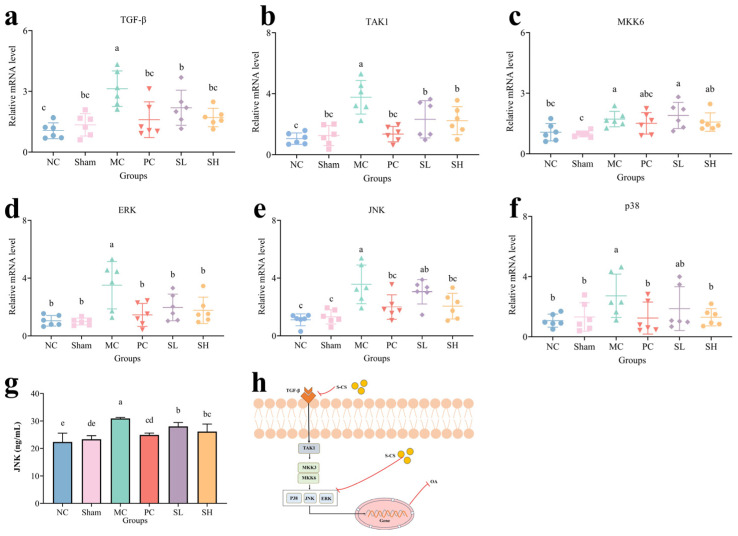
Analysis of relative mRNA levels and protein levels. (**a**) TGF-*β*, (**b**) TAK1, (**c**) MKK6, (**d**) ERK, (**e**) JNK, (**f**) p38, (**g**) The protein level of JNK, and (**h**) p38 MAPK signaling pathway diagram. (The presence of differing lowercase letters denotes significant intergroup differences, *p* < 0.05. The red lines in the mechanism diagram represent the conclusion obtained from experiments).

The levels of TGF-*β*, TAK1, ERK, JNK, and p38 (Figure 7a,b,d–f) were markedly elevated in the MC group (*p* < 0.05). TAK1 functions as a serine/threonine protein kinase responsible for initiating the MAPK pathway [65,66]. Studies have shown that CS can alleviate inflammation by inhibiting the phosphorylation of p38 MAPK, thereby potentially slowing the progression of OA [21]. Therefore, it is hypothesized that S-CS may regulate apoptosis and cartilage matrix breakdown by interfering with the activation of the p38 MAPK pathway. The mRNA level of TAK1 was significantly down-regulated in the S-CS groups (*p* < 0.05), indicating that the downregulation of TAK1 is closely related to the modulation of the MAPK signaling pathway by S-CS. Additionally, IL-1*β*-mediated JNK phosphorylation upregulates COX-2 and PGE-2 expression, contributing to ECM degradation and subsequent knee joint degeneration, resulting in chondrocyte reduction. Further analysis of the protein expression level of JNK revealed that the levels in the SH group were 26.15 ng/mL, showing significant differences relative to the MC group (30.91 ng/mL) (*p* < 0.05). It demonstrates that oral administration of S-CS significantly downregulates JNK expression levels (Figure 7g), which may be consistent with the HE staining observations. The 20 mg/kg/d S-CS group showed findings consistent with those described above, indicating that oral S-CS may suppress the MAPK pathway. Lin et al. [67] confirmed that PLM slows OA degeneration by downregulating MAPK by downregulating the protein levels of p-p38, p-JNK, and p-ERK, thereby preventing structural joint degeneration. The possible signaling pathway diagram is shown in Figure 7h.

### 3.8. Effect of S-CS on Inflammatory Cytokines

**Figure 8:**
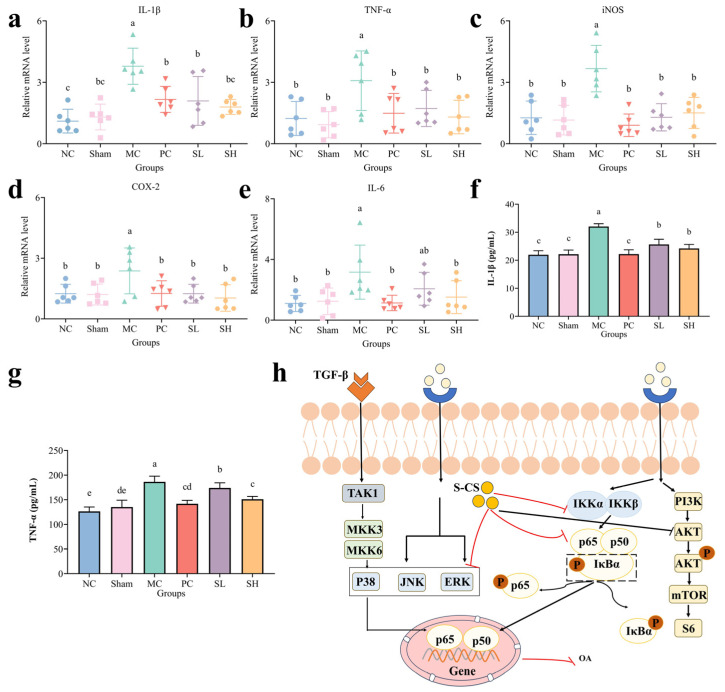
Analysis of relative mRNA levels and protein levels. (**a**) IL-1*β*, (**b**) TNF-*α*, (**c**) iNOS, (**d**) COX-2, (**e**) IL-6, (**f**) The protein level of IL-1*β*, (**g**) The protein level of TNF-*α*, and (**h**) Schematic diagram of S-CS’s effects on the signaling pathways. (The presence of different lowercase letters denotes markable intergroup differences, *p* < 0.05. The red lines in the mechanism diagram represent the conclusion obtained from experiments).

In OA, the combined action of mechanical stress and increased serum cytokines markedly disrupts cartilage homeostasis [60]. The upregulation of pro-inflammatory cytokines enhances protease activity, leading to the ECM breakdown mediated by both matrix metalloproteinases (MMPs) and aggrecanases [68]. Notably, IL-1*β* critically contributes to destabilizing cartilage integrity by impairing the equilibrium between anabolic processes (e.g., COL2A1 and ACAN synthesis) and catabolic processes (e.g., MMPs and ADAMTS-mediated degradation) within the cartilage ECM [36]. qRT-PCR analysis indicated a marked upregulation of the levels in the MC group (Figure 8a–e). After oral administration of S-CS, qPCR analysis revealed significant downregulation of IL-1*β*, TNF-*α*, iNOS, COX-2, and IL-6 expression (*p* < 0.05). IL-1*β* concentrations significantly decreased in the S-CS groups (*p* < 0.05), effectively mitigating the OA-induced increase in serum IL-1*β* concentration (Figure 8f). Serum levels of TNF-*α* were significantly reduced in the SH group by 18.94% relative to the MC group (*p* < 0.05), but in the SL group, they only decreased by 9.42%, with a clear dose-dependent trend observed among the dosage groups (Figure 8g). The possible signaling pathways are summarized in Figure 8h.

In summary, S-CS possibly exerts an osteoarthritis-alleviating effect by inhibiting the NF-*κ*B and p38 MAPK pathways and regulating inflammatory factor levels. The proposed signaling pathway is illustrated in Figure 8h.

### 3.9. Analysis of the Intestinal Microbiota

**Figure 9:**
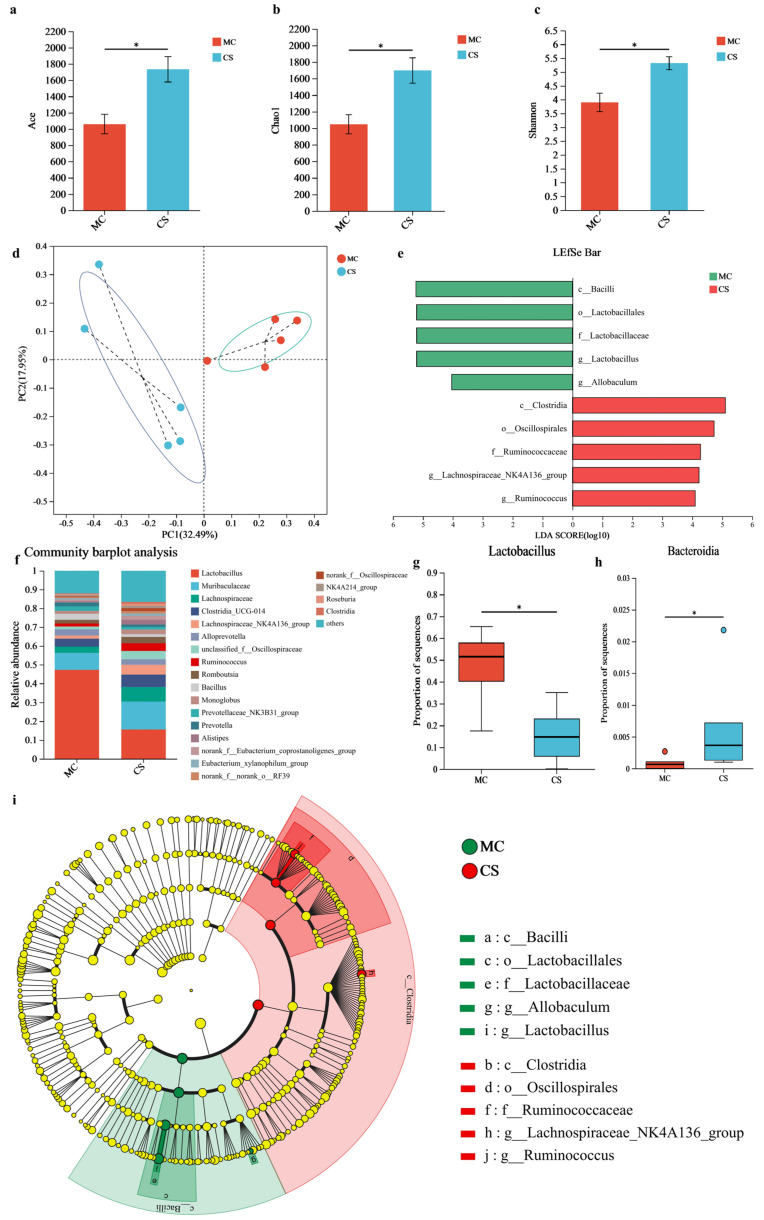
Analysis of intestinal microbiota. (**a**) Ace, (**b**) Chaol, (**c**) Shannon, (**d**) Beta diversity of gut microbiota in rats with OA by S-CS, (**e**) The distribution of dominant microbiota and the LDA threshold was 4.0, (**f**) Effect of S-CS at the genus level on the structure of the gut microbiota in OA rats, (**g**) The relative abundance of *Lactobacillus*, (**h**) The relative abundance of *Bacteroidia*, and (**i**) Cladogram displayed the taxonomic tree of differentially abundant taxa. * *p* < 0.05 vs. MC.

As shown in Figure 9a,b, a statistically significant difference was observed in the ACE and Chao 1 index (*p* < 0.05), and the Shannon alpha diversity index was also markedly elevated in the S-CS group (*p* < 0.05) (Figure 9c). PCoA analysis, which assessed the species complexity and *β*-diversity, indicated that the MC and SH groups formed two relatively distinct clusters, suggesting a clear microbial community difference between them (Figure 9d). Increased microbial diversity can improve the balance of intestinal microecology, enhance intestinal barrier function, and reduce the release of intestinal inflammatory factors, thereby alleviating joint inflammation via the gut-joint axis.

LefSe analysis identified ten key taxa as significant biomarkers at the genus level. In the MC group, five dominant microorganisms were identified: *c_Bacilli*, *o_Lactobacillales*, *f_Lactobacillaceae*, *g_Allobaculum*, and *g_Lactobacillus*, indicating a potential relationship between Lactobacillus and OA, suggesting its utility as a biomarker for OA populations (Figure 9e–g). Published evidence shows considerably greater quantities of *g_Lactobacillus* and *f_Lactobacillaceae* in individuals with OA [39,69]. Research revealed that the population of *Lactobacillus* in rheumatoid arthritis patients is significantly higher [70]. It is speculated that the high abundance of *Lactobacillus* in the MC group may be related to the abnormal activation of the NF-*κ*B pathway, which requires further mechanistic validation. In the S-CS intervention group, five dominant microbial taxa were identified, including *c_Clostridia*, *o_Oscillospirales*, *f_Ruminococcaceae*, *g_Lachnospiraceae_NK4A136_*group, and *g_Ruminococcus*. Furthermore, a CS-degrading strain of Clostridium was isolated and characterized. Notably, two human studies also reported an enhancement in the abundance of *Clostridia* following oral CS administration [71].

The selection of administration doses was determined based on preliminary experiments and relevant literature [21]. A low-dose group and a high-dose group were established in the study. Based on the observed significant differences, the high-dose group and the MC group were selected for comparison in the gut microbiota omics analysis, which is consistent with the design adopted in most omics studies. However, the use of a single dose may lead to limitations, including false-negative results, false-positive results, and incomplete dose–response relationship characterization, which should be focused on in future research.

In summary, S-CS may modulate the gut microbiota composition in OA rats. The *Lactobacillus* populations were significantly decreased in the S-CS treatment group (*p* < 0.05); conversely, the relative abundances of *Clostridia_UGG-014* and *Bacteroidia* increased. Some members of the genus *Bacteroides* secrete sulfatase, which cleaves the sulfate groups in CS and improves its utilization [71] to exert an influence on OA. These findings suggest that S-CS can effectively regulate the gut microbiota in rats to alleviate OA.

Despite these promising findings, the present study has several limitations. Although the results demonstrate that S-CS possesses a 6/4-O sulfated structure, desulfated or molecular weight-fractionated control samples were not included, leading to certain limitations in the interpretation of its structure–activity relationship. Second, differences exist between rats and adult humans in digestive function and regenerative capacity, which may affect the absorption and therapeutic efficacy of S-CS. Therefore, while the findings support the potential of S-CS as a novel functional food ingredient for alleviating OA, further validation of its efficacy and optimal dosage in human subjects is still required.

## 4. Conclusions

In this study, we characterized the S-CS, which had a GlcUA/GalN ratio of approximately 1:1 with 6-O- and 4-O-sulfation patterns. S-CS inhibited the expression of TNF-*α*, IL-6, etc., at mRNA levels, probably via regulating the NF-*κ*B and MAPK pathways. Moreover, S-CS may attenuate OA by modulating gut microbiota. These findings indicate that S-CS probably has promising potential for the prevention and treatment of OA.

## Figures and Tables

**Table 1:** Theoretical chemical shifts of ^1^H NMR for different sources of S-CS.

Residues	Proton	^1^H Chemical Shift (ppm)
GlcUA	UH-1	4.49
	UH-2	3.37
	UH-3	3.58
	UH-4	3.70–3.79
	UH-5	3.70–3.79
GalNAc	NH-1	4.54
	NH-2	4.02
	NH-3	4.18
	NH-4	4.22
	Nac4S (CH3)	2.04
	Nac6S (CH3)	2.02

**Table 2:** Theoretical chemical shifts of ^13^C NMR for different sources of S-CS.

Residues	^13^C Chemical Shift (ppm)
GlcUA-C_1_-GalNAc6S	107.22
GlcUA-C_1_-GalNAc4S	106.68
GlcUA-C_2_	75.41
GlcUA-C_3_	76.65
GlcUA-C_4_	83.38
GlcUA-C_5_	79.32
GalNAc6S-C_1_	104.51
GalNAc4S-C_1_	103.78
GalNAc4S-C_2_	54.53
GalNAc6S-C_2_	53.80
GalNAc-C_3_	82.98
GalNAc-C_5_	75.34
GalNAc6S-C_4/6_	70.61
GalNAc4S-C_6_	64.05
GalNAc-CH_3_	25.31

**Table 3:** Related data of the subchondral bone of the knee joint of rats.

Subchondral Bone	BS/BV	BS/TV	Tb.N	Tb.Sp	BMD
NC	17.53046	9.73648	3.11226	0.18551	0.65107
Sham	18.25876	9.38568	2.76274	0.19041	0.63301
MC	14.49734	8.07698	2.35623	0.19875	0.66514
PC	15.16292	8.62569	2.58438	0.19127	0.68420
SL	13.44116	7.94689	2.27407	0.19400	0.64446
SH	17.60255	9.32476	2.71791	0.16868	0.71008

## Data Availability

The original contributions presented in the study are included in the article/Supplementary Materials; further inquiries can be directed to the corresponding author.

## Supplementary Materials

The following supporting information can be downloaded at: https://www.mdpi.com/article/10.3390/nu18101494/s1, Table S1: Specific primer sequences.
