# Supplementary material for: Structural Characterization of Chondroitin Sulfate from Hybrid Sturgeon (Acipenser schrenckii × Huso dauricus) Cartilage and Its Alleviating Effect on Osteoarthritis
Source: Nutrients. 2026 May 8;18(10):1494. doi: 10.3390/nu18101494 (PMC13209495; doi:10.3390/nu18101494)
Supplement: Supplementary file 1 [file nutrients-18-01494-s001.zip › nutrients-4223123-supplementary.pdf]

Table S1. qPCR primer sequences.

Gene	Forward	Reverse
ERK	AGGTCAGTGCCACCATGAGGAG	GCCATAGCCAGCCACGGTTC
IκBα	TCACCAACCAGCCAGGAATTGC	TGCTCCACGATGCCCAGGTAG
p50	AAGAACAGCAAGGCAGCACTCC	AGGTGTCGTCCCATCGTAGGTG
IKKβ	GGAGCAGACGGAGTTTGGCATC	TCCTGGCTGTCACCTTCTGTCC
IKKα	TCGCAAAGTGTGGGCTGAAGC	CTCCCTGGCGTCTCCCATAGG
p65	CCGACGTATTGCTGTGCCTTCC	CTGCCTGGGTGCTCTTTGGAAC
iNOS	ACCAGAGGACCCAGAGACAAGC	GCCGACCTGATGTTGCCACTG
COX-2	TGTATCCCGCCCTGCTGGTG	TGCGTTGATGGTGGCTGTCTTG
MKK6	CATGGCGGTGAAGCGGATACG	TTGGCCCAGCGTGTTAATGAGC
TGF-β	GCAGTGGCTGAACCAAGGAGAC	CTGGAGCTGTGCAGGTGTTGAG
JNK	AGCTCTCCAGCACCCGTACATC	CTACAGCAGCCCAGAGGTCCAG
TNF-α	TCCAGAACTCCAGGCGGTGTC	TGGGCTACGGGCTTGTCACTC
p38	GGGACATCGTGTGGCAGTGAAG	GCGAGGTTGCTGGGCTTTAGG
TAK1	GGGAGTGCTGCTTGGATGGC	CCTGTGCTGGTGGCTGAGTTG
IL-1β	CGTGCTGTCTGACCCATGTGAG	TGTCCCGACCATTGCTGTTTCC
IL-6	TGCTCTGGTCTTCTGGAGTTCC	AGGTTTGCCGAGTAGACCTCA
